# Health-related quality of life is inversely correlated with C-reactive protein and age in *Mycobacterium avium* complex lung disease: a cross-sectional analysis of 235 patients

**DOI:** 10.1186/s12931-015-0304-5

**Published:** 2015-12-03

**Authors:** Takanori Asakura, Yohei Funatsu, Makoto Ishii, Ho Namkoong, Kazuma Yagi, Shoji Suzuki, Takahiro Asami, Tetsuro Kamo, Hiroshi Fujiwara, Yoshifumi Uwamino, Tomoyasu Nishimura, Sadatomo Tasaka, Tomoko Betsuyaku, Naoki Hasegawa

**Affiliations:** Division of Pulmonary Medicine, Department of Medicine, Keio University School of Medicine, 35 Shinanomachi, Shinjuku-ku, Tokyo 160-8582 Japan; Center for Infectious Diseases and Infection Control, Keio University School of Medicine, Tokyo, Japan; Keio University Health Center, Tokyo, Japan

**Keywords:** C-reactive protein, *Mycobacterium avium* complex, Nontuberculous mycobacteria, Quality of life

## Abstract

**Background:**

*Mycobacterium avium* complex (MAC) lung diseases generally cause chronic disease in immunocompetent hosts. Although a few studies have examined health-related quality of life (HRQL) in patients with MAC lung disease, there have been no large studies. This study aimed to evaluate HRQL and its correlation with clinical outcomes in MAC lung disease.

**Methods:**

A cross-sectional study was conducted at Keio University Hospital to investigate the factors associated with HRQL in pulmonary nontuberculous mycobacterial diseases. MAC lung diseases were diagnosed according to the 2007 ATS/IDSA guidelines for nontuberculous mycobacterial diseases. The 36-item short form health survey (SF-36) was administered to assess clinical outcomes. Clinical variables included treatment status, latest haematological data, and bacterial smear and culture results.

**Results:**

The SF-36 scores for the 235 patients (median age, 69 years; 45 men and 190 women) with MAC lung disease, except for the bodily pain and mental health subscale scores, were significantly lower than the Japanese population norms. In the multivariable analyses, current treatment for MAC and a positive sputum smear or culture within the past year were significantly associated with lower SF-36 scores. C-reactive protein (CRP) and age showed stronger inverse correlations with SF-36 scores.

**Conclusions:**

HRQL, especially the physical component, was impaired in patients with MAC lung diseases; this appears to be related with current treatment status, positive sputum smear or culture within the previous year, and particularly CRP and age. Further studies including qualitative assessments are needed to investigate the efficacy of CRP as a marker for progression or treatment response in MAC lung disease.

**Trial registration:**

Clinical trial registered with UMIN (UMIN000007964).

## Background

The incidence of nontuberculous mycobacterial lung disease is increasing worldwide [1, 2]. *Mycobacterium avium* complex (MAC) lung disease, the most common nontuberculous mycobacterial disease, generally causes chronic, slowly progressive disease in immunocompetent hosts [3].

Therapy involving multiple antimicrobials against MAC is generally effective because it decreases the bacterial load, resulting in a change from a positive to negative sputum culture. However, established MAC lung diseases are often incurable or recurrent, resembling other chronic diseases such as diabetes mellitus (DM), chronic obstructive pulmonary disease (COPD), or interstitial lung disease (ILD). Long-term treatment with multiple antimicrobial agents and their side effects frequently contribute to patient burden.

As an indicator of overall health status, patient-reported outcome measures representing health-related quality of life (HRQL), or the individual’s satisfaction or happiness with aspects of life related to health, have become important in the current treatment guidelines for MAC lung disease, in addition to conventional measures of infection control or cure rate [3, 4]. Furthermore, HRQL provides information for health management and policy decisions [5]. The 36-item Short-Form health survey (SF-36) version 2 is a general HRQL measure and has been used in different chronic respiratory diseases, including ILD [6], sarcoidosis [7], bronchiectasis [8], pulmonary tuberculosis [9], and COPD [10]. With MAC lung disease, only two studies reported impaired HRQL, as assessed using the SF-36 and St. George’s Respiratory Questionnaire (SGRQ) [11, 12], a respiratory disease-specific HRQL instrument originally designed for patients with COPD [13]. However, the correlations between HRQL and variables such as treatment status, sputum culture and smear results, and haematological data remain unknown.

This study aimed to evaluate HRQL, as assessed using the SF-36, and identify its clinical determinants in a large population of consecutive patients with MAC lung disease.

## Methods

### Study population

A cross-sectional study was conducted at Keio University Hospital with patients with pulmonary nontuberculous mycobacterial diseases (University Hospital Medical Information Network: UMIN000007964). The study protocol was approved by the Keio University Hospital ethics review board. Written informed consent was obtained from each patient.

We enrolled 285 patients aged ≥20 years who were diagnosed with nontuberculous mycobacterial diseases between May 2012 and February 2014 according to the 2007 American Thoracic Society/Infectious Disease Society of America guidelines for the diagnosis of nontuberculous mycobacterial diseases [3]. We excluded 31 patients who could not complete the HRQL questionnaire and 19 patients with nontuberculous mycobacterial diseases other than MAC. The final sample included 235 patients with MAC lung disease.

### Health-related quality of life assessment

All patients completed the SF-36 version 2 [14] and SGRQ in Japanese [13, 15, 16]. The SF-36 consists eight subscales: physical functioning, role-physical, bodily pain, general health, vitality, social functioning, role-emotional, and mental health. These eight subscales provide three summary scores, which were adjusted for Japanese patients: physical, mental, and role/social component summary scores [17]. All scores were transformed to fit a norm-based score for the general Japanese population, with a mean score of 50 and standard deviation of 10. Lower scores indicate poorer HRQL. The internal consistency of the SF-36 was assessed using Cronbach’s α.

SGRQ scores (range, 0–100) were calculated for the total questionnaire as well as for each of the three components; the symptoms, activity, and impact components measure respiratory symptoms, impairment of physical activity, and social and psychological disturbances, respectively.

### Assessment of clinical parameters

Demographic data included age at diagnosis, disease duration, body mass index (BMI), smoking status, underlying pulmonary diseases, comorbidities using the age-adjusted Charlson comorbidity index (CCI) [18], treatment status, and bacterial smear and culture results for *M. avium* and *M. intracellulare* at study enrolment. Haematological investigations and pulmonary function tests were performed after study enrolment. Treatment status was classified as never treated, previously treated, or currently treated. The lower respiratory tract specimens were cultured on egg-based solid media (Kyokuto Pharmaceutical Industrial Co., Ltd., Tokyo, Japan) or mycobacteria growth indicator tubes (Becton, Dickinson and Co., Sparks, MD, USA). The AccuProbe system (Gen-Probe Inc., San Diego, CA, USA) was used to identify MAC isolates. The COBAS AMPLICOR system (Roche Diagnostic Co., Ltd., Tokyo, Japan) and DNA–DNA hybridization test (Kyokuto Pharmaceutical Industrial Co., Ltd.) were used to classify MAC isolates as *M. avium* or *M. intracellulare*; unspecified MAC isolates were categorized as MAC. Sputum smears or cultures were defined as negative based on results obtained in the previous year. If patients did not cough up sputum, they were recorded as negative [19]. Pulmonary function tests were performed with patients with a stable condition using an electronic spirometer according to the American Thoracic Society guidelines [20].

### Statistical analyses

Correlations between two continuous variables were analysed using Spearman’s correlation coefficients. Comparisons were conducted using Student’s *t*-tests between two groups and Tukey's tests among three groups. Analyses of covariance (ANCOVA), adjusted for age, sex, CCI, smoking status, BMI, and underlying pulmonary disease, were used to analyse group differences. To identify the relative contributions of factors to SF-36 scores lower than the Japanese population norms, factors significantly associated with lower norm-based SF-36 scores in the univariate analyses were entered into a stepwise forward and backward multiple regression model for multivariate analysis. All *P* values were two-tailed; *P* < 0.05 was considered significant. Statistical analyses were conducted using JMP v11.0 (SAS Institute Japan Ltd, Tokyo, Japan).

## Results

### Patient characteristics and clinical features

The median (interquartile range) age of the patients was 69 (64–76) years; 190 (80.9 %) patients were women, and 215 (91.4 %) patients had never smoked (Table 1). Mean BMI was 19.5 (17.5–21.2) kg/m^2^. *M. intracellulare* (5.1 %) was the least commonly isolated bacteria. Underlying pulmonary diseases were present in 36 (15.3 %) patients. Regarding treatment status, 110 (46.8 %) patients were never treated, 80 (34.0 %) were previously treated, and 45 (19.1 %) were currently treated. Smear tests or sputum cultures were positive for MAC in 78 (33.2 %) and 132 (56.5 %) patients, respectively. The pulmonary function test results were within the normal range; percent-predicted functional volume capacity (%FVC), percent-predicted forced expiratory volume in 1 s, and percent-predicted diffusing capacity of the lung for carbon monoxide (%DL_CO_) were 95.7 (interquartile range, 80.1–108.0), 87.3 (interquartile range, 72.7–99.1), and 88.7 (interquartile range, 78.2–104.6), respectively.

**Table 1:** Clinical characteristics of patients with *Mycobacterium avium* comlex lung disease (*n* = 235)

	Median (interquartile range) or number (%)
Age, years	69 (64–76)
Disease duration, years	5 (3–10)
Sex, female	190 (80.8)
Body mass index, kg/m^2^	19.5 (17.5–21.2)
Mycobacterium species	
*Mycobacterium avium*	128 (54.5)
*Mycobacterium intracellulare*	12 (5.1)
MAC	95 (40.4)
Smoking status	
Never	215 (91.5)
Former	20 (8.5)
Current	0 (0)
Charlson comorbidity index	3 (3–4)
Underlying pulmonary diseases	
Asthma	8 (3.4)
COPD	2 (0.9)
Interstitial lung disease	1 (0.4)
Lung cancer	2 (0.9)
Old pulmonary TB	23 (9.8)
Treatment status	
Never treated	110 (46.8)
Previously treated	45 (19.1)
Currently treated	80 (34.0)
Bacterial status in the previous year	
Positive smear	78 (33.2)
Positive culture	132 (56.1)
%FVC, %	95.7 (80.1–108.0)
%FEV_1_, %	87.3 (72.7–99.1)
%DL_CO_, %	88.7 (78.2–104.6)

*MAC Mycobacterium avium* complex, *COPD* chronic obstructive pulmonary disease, *TB* tuberculosis, *FVC* functional volume capacity, *FEV*_*1*_ forced expiratory volume in 1 s, *DL*_*CO*_ diffusing capacity of the lung for carbon monoxide

### Norm-based SF-36 scores

**Fig. 1:**
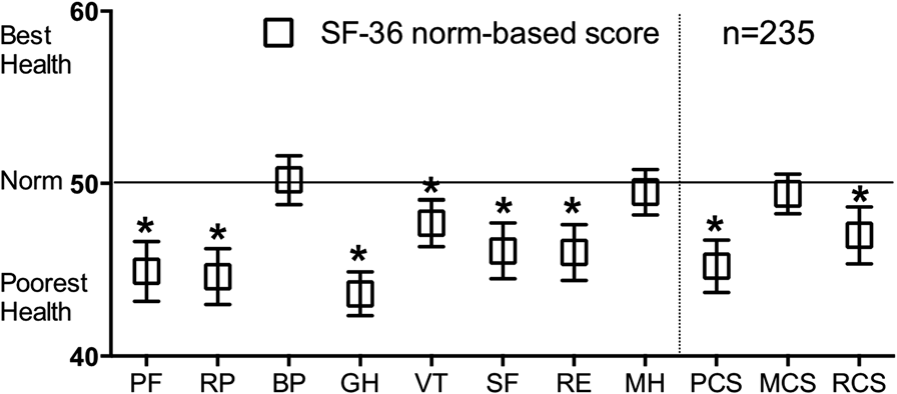
Norm-based SF-36 scores in patients with MAC lung disease. Error bars represent the 95 % confidence intervals. Higher SF-36 scores are related with better health-related quality of life (HRQL). *Scores <50 indicate poorer HRQL than the Japanese population norms. SF-36: 36-item short form healthy survey; MAC, *Mycobacterium avium* complex; PF, physical functioning; RP, role - physical; BP, bodily pain; GH, general health; VT, vitality; SF, social functioning; RE, role-emotional; MH, mental health; PCS, physical component summary; MCS, mental component summary; RCS, role/social component summary

The norm-based SF-36 scores are shown in Fig. 1. Of the subscale components, the bodily pain, and mental health scores were within the range of the Japanese population norms; however, the physical functioning, role-physical, general health, vitality, social functioning, and role-emotional subscale scores were significantly lower than the Japanese population norms. The general health subscale represented the largest difference (difference, 6.4; 95 % CI, 5.1–7.7). For the summary scores, the physical and role/social component summary scores were significantly lower than the Japanese population norms. The physical component summary score represented the largest difference (difference, 4.8; 95 % CI, 3.3–6.3).

### Correlations between SF-36 subscale or component summary scores and clinical parameters, physiological parameters, and SGRQ scores

The internal consistency of the SF-36 was good (Cronbach’s α, 0.90–0.92). The correlations between the SF-36 subscale or component summary scores and pulmonary function tests and SGRQ scores are shown in Table 2. All SF-36 subscale scores were significantly correlated with each other and with the component summary scores. All SF-36 subscale or component summary scores had significant and moderate to strong correlations with all SGRQ scores.

**Table 2:**
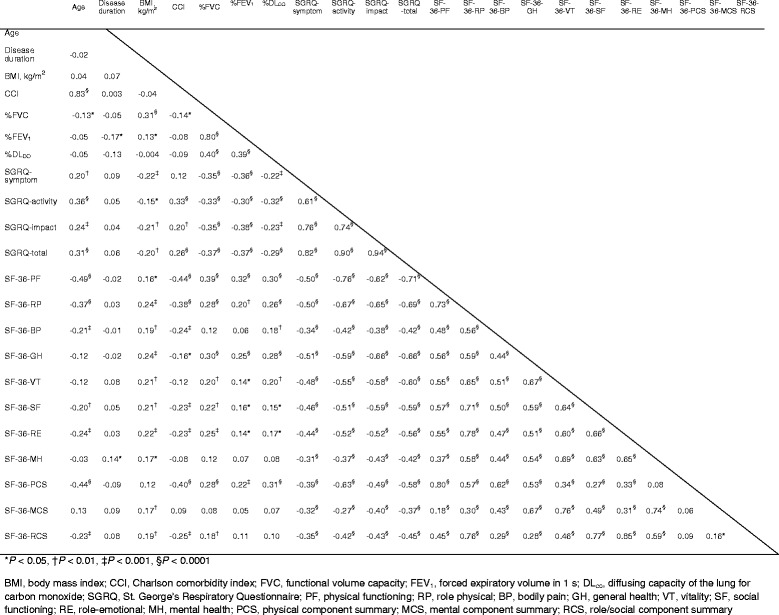
Spearman’s correlations between the SF-36 scores and clinical parameters in patients with MAC lung disease (*n* = 235)

Pulmonary function showed stronger correlations with the physical functioning, role-physical, and general health subscale scores and the physical component summary score than the other subscale scores or component summary scores. Age was significantly correlated with all SF-36 scores except the general health, vitality, and mental health subscale scores and the mental component summary score; the disease duration was poorly correlated with all SF-36 scores. BMI had a significant but weak correlation with the SF-36 scores, excluding the physical component summary score. Age-adjusted CCI had a significant and moderate correlation with the physical functioning and role-physical subscale scores and the physical component summary scores.

### Correlations between SF-36 subscale or component summary scores and haematological data

Correlations between the SF-36 subscale or component summary scores and haematological data are shown in Table 3. Haemoglobin (Hb) and C-reactive protein (CRP) levels were significantly correlated with the physical functioning, role-physical, general health, vitality, social functioning, and role-emotional subscale scores and the physical and role/social component summary scores; CRP was strongly correlated with the physical functioning subscale score. Alkaline phosphatase, sialylated carbohydrate antigen KL-6, and white blood cell count were moderately correlated with physical component summary score. Hb was moderately correlated with the physical functioning and role-physical subscale scores and physical component summary scores.

**Table 3:** Spearman’s correlations between SF-36 scores and haematological data in patients with MAC lung disease (*n* = 235)

	Median (interquartile range)	PF	RP	BP	GH	VT	SF	RE	MH	PCS	MCS	RCS
TP, g/dL	6.9 (6.7–7.3)	−0.11	−0.06	−0.34	−0.13	−0.31	0.06	0.04	−0.02	−0.16	−0.04	0.10
Creatinine, mg/dL	0.66 (0.58–0.76)	0.05	0.10	0.15	0.15	0.17*	0.12	0.08	0.09	0.06	0.18*	0.02
LDH, IU/L	179 (162–199)	−0.16	−0.08	−0.05	−0.05	−0.08	−0.09	−0.04	−0.09	−0.09	−0.04	−0.07
ALP, IU/L	230 (187–289)	−0.25**	−0.21*	−0.12	−0.19**	−0.12	−0.10	−0.19*	−0.11	−0.24**	−0.04	−0.11
KL-6, U/mL	251 (194–393)	−0.22**	−0.26***	−0.09	−0.20*	−0.16	−0.12	−0.19*	−0.10	−0.19*	−0.06	−0.13
WBC, /μL	5600 (4500–6400)	−0.17*	−0.15	0.00	−0.13	−0.07	−0.01	−0.10	−0.002	−0.21**	0.06	−0.04
Haemoglobin, g/dL	13.0 (12.1–13.7)	0.30***	0.23**	0.14	0.18*	0.19*	0.24**	0.23**	0.09	0.22**	0.003	0.20*
Platelets, /μL	21.3 (18.0–24.9)	−0.005	−0.001	0.07	−0.02	0.01	0.01	0.01	0.02	−0.02	0.02	0.03
CRP, mg/dL	0.06 (0.02–0.33)	−0.35***	−0.30***	−0.13	−0.20*	−0.18*	−0.26***	−0.24**	−0.10	−0.28***	0.02	−0.23**
SPD, ng/mL	78 (54–12)	−0.19*	−0.22**	−0.10	−0.03	−0.06	−0.10	−0.17*	−0.06	−0.13	0.03	−0.16
GPL-IgA core, U/mL	1.5 (0.5–7.2)	−0.10	−0.13	−0.10	−0.11	−0.19*	−0.12	−0.14	−0.13	−0.04	−0.13	−0.10

*SF-36* 36-item short form healthy survey, *MAC Mycobacterium avium* complex, *PF* physical functioning, *RP* role physical, *BP* bodily pain, *GH* general health, *VT* vitality, *SF* social functioning, *RE* role-emotional, *MH* mental health, *PCS* physical component summary, *MCS* mental component summary, *RCS* role/social component summary, *TP* total protein, *LDH* lactate dehydrogenase, *ALP* alkaline phosphatase, *KL-6* sialylated carbohydrate antigen KL-6, *WBC* white blood cell, *CRP* C-reactive protein, *SPD* surfactant protein d, *GPL-IgA core* glycopeptidolipid core immunoglobulin A antibody

*P < 0.01, **P < 0.001, ***P < 0.0001

### Comparisons between SF-36 subscale or component summary scores using ANCOVA

**Fig. 2:**
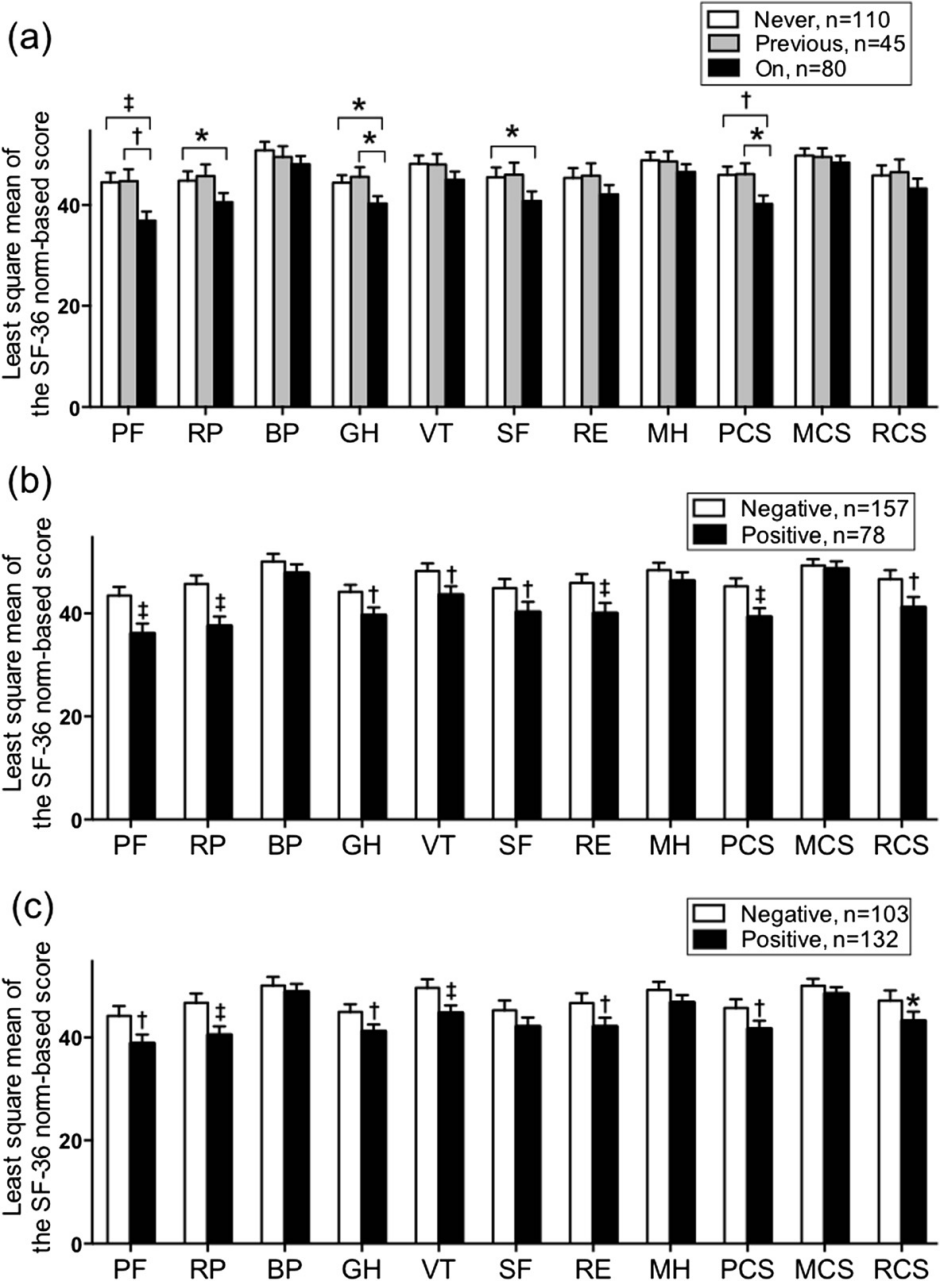
Results of the comparisons of SF-36 subscale or component summary scores. Displayed as least square means, based on (**a**) treatment status, (**b**) sputum smear, and (**c**) sputum culture after adjustment for age, sex, Charlson comorbidity index, smoking status, body mass index, and underlying pulmonary disease in patients with pulmonary *Mycobacterium avium* complex lung diseases. Error bars represent the standard error of the mean. Comparisons were conducted between two groups using Student *t*-tests and among three groups using Tukey's tests. *P < 0.05, ^†^ *P* < 0.01, ^‡^ *P* < 0.001. SF-36: 36-item short form healthy survey; PF, physical functioning; RP, role physical; BP, bodily pain; SF, social functioning; GH, general health; VT, vitality; RE, role-emotional; MH, mental health; PCS, physical component summary; MCS, mental component summary; RCS, role/social component summary

The physical functioning, role-physical, general health, and social functioning subscale and physical component summary scores significantly differed between the never- and currently-treated groups and between the previously- and currently-treated groups, but not between the never- and previously-treated groups (Fig. 2a). The physical functioning, role-physical, general health, vitality, and role-emotional subscale scores and the physical and role/social component summary scores significantly differed by sputum smear or culture results (Fig. 2b, c).

### Multivariate determinants for SF-36 subscale or component summary scores

The stepwise multiple regression analysis included age, sex, age-adjusted CCI, BMI, smoking status, disease duration, underlying pulmonary diseases, treatment status (currently treated vs previously/never treated), positive sputum smear or culture, pulmonary function, Hb levels, and CRP levels (Table 4). CRP levels, age, %FVC, underlying pulmonary disease, %DL_CO_, current treatment, and sputum culture significantly predicted physical functioning subscale score, accounting for 56.0 % of the variance. CRP levels, age, sputum culture, and %DL_CO_ significantly predicted role-physical subscale scores, accounting for 34.5 % of the variance. %FVC, age, CRP levels, and %DL_CO_ significantly predicted general health subscale score, accounting for 26.8 % of the variance. %FVC, CRP level, and sputum culture significantly predicted the vitality subscale score, accounting for 17.6 % of the variance. CRP level, age, and %FVC significantly predicted social functioning and role-emotional subscale scores, accounting for 26.5 % and 18.7 % of the variance, respectively. Regarding the physical component summary score, age, CRP levels, %DL_CO_, sputum culture, and underlying pulmonary diseases were significant predictors, accounting for 47.4 % of the variance. CRP levels and age were significant predictors for almost all SF-36 scores that were lower than the Japanese population norms.

**Table 4:** Multivariate determinants for SF-36 scores lower than the Japanese population norms (*n* = 235)

SF-36		*P*-value	Cumulative R^2^ (%)
PF	CRP	<0.0001	26.1
	Age	<0.0001	38.7
	%FVC	0.0002	44.9
	UPD	0.0008	49.5
	%DL_CO_	0.0045	52.5
	Current treatment	0.0206	54.5
	Sputum culture	0.0441	56.0
RP	CRP	<0.0001	15.3
	Age	0.0001	25.3
	Sputum culture	0.0007	31.6
	%DL_CO_	0.0110	34.5
GH	%FVC	<0.0001	13.1
	Age	0.0009	20.1
	CRP	0.0076	24.4
	%DL_CO_	0.0397	26.8
VT	%FVC	0.0006	8.6
	CRP	0.0093	13.2
	Sputum culture	0.0100	17.6
SF	CRP	<0.0001	18.8
	Age	0.0062	23.3
	%FVC	0.0202	26.5
RE	CRP	0.0002	9.8
	%FVC	0.0068	14.7
	Age	0.0122	18.7
PCS	Age	<0.0001	21.6
	CRP	<0.0001	34.9
	%DL_CO_	0.0003	41.1
	Sputum culture	0.0125	43.9
	UPD	0.0290	47.4
RCS	CRP	0.0001	10.4
	Age	0.0345	13.4

*SF-36* 36-item short form healthy survey, *PF* physical functioning, *CRP* C-reactive protein, *FVC* functional volume capacity, *UPD* underlying pulmonary disease, *DL*_*CO*_ diffusing capacity of the lung for carbon monoxide, *RP* role physical, *GH* general health, *SF* social functioning, *RE* role-emotional, *PCS* physical component summary, *RCS* role/social component summary

## Discussion

Based on this study of HRQL in patients with MAC lung disease and the correlations between SF-36 and clinical factors, there were three important findings. First, patients with MAC lung disease have significantly poorer HRQL than the Japanese population norms, implying impaired physical performance and social activities. Second, we identified two clinically important factors associated with lower HRQL: currently receiving treatment and positive sputum smear or culture within the previous year. Finally, we also identified several factors, including CRP levels and age that were strongly associated with poorer HRQL.

A number of previous studies have demonstrated impaired HRQL in various chronic diseases, including DM; patients with DM had lower scores for all eight SF-36 subscales compared with US population norms [21] and a higher risk for low physical component summary scores compared with older Japanese adults without DM [22]. In stroke patients, neurological impairments were associated with functional disability shortly after the stroke; lower physical and mental component summary scores were present at 3 months and 1 year after the stroke [23]. In addition, in the general population, common chronic conditions (e.g., ischemic heart disease, DM, and arthritis) notably impacted the physical functioning, general health, and bodily pain subscales, respectively, in a previous study. Furthermore, lower SF-36 physical component summary scores have been reported for patients with arthritis, chronic lung disease, or congestive heart failure [24]. The severity of chronic lung diseases, including chronic bronchitis, emphysema, and asthma, are reportedly significantly correlated with all eight SF-36 scales, reflecting respiratory symptoms [25, 26]; the physical component summary score is primarily affected in COPD [27], asthma [28], ILD [6], and sarcoidosis [7], while bronchiectasis impacts both the physical and mental component summary scores [8].

To the best of our knowledge, only two studies have evaluated HRQL in patients with MAC lung disease. Impaired SGRQ-assessed HRQL was associated with high-resolution computed tomography findings [12]. Furthermore, in a study based in Canada, all of the subscale and component summary scores were impaired [11]; comparatively, the bodily pain and mental health subscale scores, and the mental component summary scores were within the population norm ranges in the present study. Pulmonary function was better, and there were fewer patients being currently treated in the present study, which might explain the differences in the results, in addition to differences in nationality or race.

In the present study, there were differences in HRQL, as evaluated using SF-36, between the treatment statuses. Patients currently receiving medical treatment had lower HRQL than the other treatment groups, while there were no differences in HRQL between the never- and previously-treated groups. These results suggest that multiple antimicrobial therapies against MAC might have a significant negative impact on HRQL, although patients who are currently receiving treatment tend to have more severe MAC lung disease. Sputum status, especially in culture conversion, has been used classically to evaluate infection control in MAC lung disease [29–31]. Notably, our data indicate the ability of sputum status to measure HRQL in patients with MAC lung disease.

To the best of our knowledge, our study is the first to demonstrate strong correlations between SF-36 scores and multiple clinical factors including patient characteristics, sputum culture status, pulmonary function, and haematological data. Age has already been reported as an important factor negatively influencing the SF-36 score [32]; given the aging population, this correlation is likely to become more important [33]. Furthermore, our study revealed that CRP might reflect HRQL-associated disease activity in patients with MAC lung disease. CRP is a biomarker that is elevated in acute and chronic inflammation such as that in infectious disease or non-infectious chronic diseases. Previous studies in chronic disease have demonstrated the ability of CRP to predict cardiovascular events in patients with arteriosclerotic diseases [34], treatment failure in tuberculosis [35] or human immunodeficiency virus infection [36], and disease progression in ILD with systemic scleroderma [37]. CRP might be a better and more convenient marker than a physiological parameter and could potentially be a marker of disease progression or treatment response.

Our study has several potential limitations. First, the cross-sectional design creates challenges in determining causal relationships, particularly regarding the influence of treatment. Second, by including only patients who could complete the SF-36, SGRQ, and pulmonary function tests, we might have excluded patients with more severe disease. However, we believe the present findings also apply to patients with severe disease because the pulmonary function and some of the haematological results were significantly correlated with HRQL.

## Conclusions

In conclusion, patients with MAC lung disease have significantly lower HRQL than the Japanese population norms, indicating impaired physical performance and social activities. These impairments appear to be related with current treatment status and positive sputum smear or culture within the previous year. CRP levels and age were the most significant factors for impaired HRQL. These findings could affect treatment initiation decisions by physicians. Further studies including qualitative assessments are needed to investigate the efficacy of CRP as a marker for progression or treatment response in MAC lung disease.

## Abbreviations

%DL_CO_: percent - predicted diffusing capacity of the lung for carbon monoxide
%FVC: percent-predicted functional volume capacity
ANCOVA: analyses of covariance
BMI: body mass index
CCI: Charlson comorbidity index
CI: confidence interval
COPD: chronic pulmonary obstructive disease
CRP: C-reactive protein
DM: diabetes mellitus
Hb: haemoglobin
HRQL: health-related quality of life
ILD: interstitial lung disease
MAC: *Mycobacterium avium* complex
SF-36: 36-item short form healthy survey
SGRQ: St. George’s Respiratory Questionnaire

## References

[1] Satta G, McHugh TD, Mountford J, Abubakar I, Lipman M (2014). Managing pulmonary nontuberculous mycobacterial infection. time for a patient-centered approach. Ann Am Thorac Soc.

[2] Kendall BA, Winthrop KL (2013). Update on the epidemiology of pulmonary nontuberculous mycobacterial infections. Semin Respir Crit Care Med..

[3] Griffith DE, Aksamit T, Brown-Elliott BA, Catanzaro A, Daley C, Gordin F (2007). An official ATS/IDSA statement: diagnosis, treatment, and prevention of nontuberculous mycobacterial diseases. Am J Respir Crit Care Med..

[4] Management of opportunist mycobacterial infections: Joint Tuberculosis Committee Guidelines 1999. Subcommittee of the Joint Tuberculosis Committee of the British Thoracic Society. Thorax. 2000;55:210–8. 10.1136/thorax.55.3.210 PMC1745689 10679540

[5] Guyatt GH, Feeny DH, Patrick DL (1993). Measuring health-related quality of life. Ann Intern Med..

[6] Chang JA, Curtis JR, Patrick DL, Raghu G (1999). Assessment of health-related quality of life in patients with interstitial lung disease. Chest..

[7] Cox CE, Donohue JF, Brown CD, Kataria YP, Judson MA (2004). Health-related quality of life of persons with sarcoidosis. Chest..

[8] Guilemany JM, Alobid I, Angrill J, Ballesteros F, Bernal-Sprekelsen M, Picado C (2006). The impact of bronchiectasis associated to sinonasal disease on quality of life. Respir Med..

[9] Dion MJ, Tousignant P, Bourbeau J, Menzies D, Schwartzman K (2004). Feasibility and reliability of health-related quality of life measurements among tuberculosis patients. Qual Life Res..

[10] Miyazaki M, Nakamura H, Chubachi S, Sasaki M, Haraguchi M, Yoshida S (2014). Analysis of comorbid factors that increase the COPD assessment test scores. Respir Res..

[11] Mehta M, Marras TK (2011). Impaired health-related quality of life in pulmonary nontuberculous mycobacterial disease. Respir Med..

[12] Maekawa K, Ito Y, Oga T, Hirai T, Kubo T, Fujita K (2013). High-resolution computed tomography and health-related quality of life in Mycobacterium avium complex disease. Int J Tuberc Lung Dis..

[13] Jones PW, Quirk FH, Baveystock CM, Littlejohns P (1992). A self-complete measure of health status for chronic airflow limitation. The St. George's Respiratory Questionnaire. Am Rev Respir Dis.

[14] Fukuhara S, Bito S, Green J, Hsiao A, Kurokawa K (1998). Translation, adaptation, and validation of the SF-36 Health Survey for use in Japan. J Clin Epidemiol..

[15] Hajiro T, Nishimura K, Tsukino M, Ikeda A, Koyama H, Izumi T (1998). Comparison of discriminative properties among disease-specific questionnaires for measuring health-related quality of life in patients with chronic obstructive pulmonary disease. Am J Respir Crit Care Med..

[16] Hajiro T, Nishimura K, Tsukino M, Ikeda A, Koyama H, Izumi T (1998). Analysis of clinical methods used to evaluate dyspnea in patients with chronic obstructive pulmonary disease. Am J Respir Crit Care Med..

[17] Suzukamo Y, Fukuhara S, Green J, Kosinski M, Gandek B, Ware JE (2011). Validation testing of a three-component model of Short Form-36 scores. J Clin Epidemiol..

[18] Charlson M, Szatrowski TP, Peterson J, Gold J (1994). Validation of a combined comorbidity index. J Clin Epidemiol..

[19] Kobashi Y, Matsushima T, Oka M (2007). A double-blind randomized study of aminoglycoside infusion with combined therapy for pulmonary Mycobacterium avium complex disease. Respir Med..

[20] Standardization of Spirometry, 1994 Update. American Thoracic Society. Am J Respir Crit Care Med. 1995;152:1107–36. doi:10.1164/ajrccm.152.3.7663792. 10.1164/ajrccm.152.3.7663792 7663792

[21] Norris SL, McNally TK, Zhang X, Burda B, Chan B, Chowdhury FM (2011). Published norms underestimate the health-related quality of life among persons with type 2 diabetes. J Clin Epidemiol..

[22] Nezu S, Okamoto N, Morikawa M, Saeki K, Obayashi K, Tomioka K (2014). Health-related quality of life (HRQOL) decreases independently of chronic conditions and geriatric syndromes in older adults with diabetes: the Fujiwara-kyo Study. J Epidemiol..

[23] Ayis S, Wellwood I, Rudd AG, McKevitt C, Parkin D, Wolfe CD (2015). Variations in Health-Related Quality of Life (HRQoL) and survival 1 year after stroke: five European population-based registers. BMJ Open..

[24] Alonso J, Ferrer M, Gandek B, Ware JE, Aaronson NK, Mosconi P (2004). Health-related quality of life associated with chronic conditions in eight countries: results from the International Quality of Life Assessment (IQOLA) Project. Qual Life Res..

[25] Selim AJ, Ren XS, Fincke G, Rogers W, Lee A, Kazis L (1997). A symptom-based measure of the severity of chronic lung disease: results from the Veterans Health Study. Chest..

[26] Ruffin RE, Wilson DH, Chittleborough CR, Southcott AM, Smith B, Christopher DJ (2000). Multiple respiratory symptoms predict quality of life in chronic lung disease: a population-based study of Australian adults. Qual Life Res..

[27] van Manen JG, Bindels PJ, Dekker FW, Bottema BJ, van der Zee JS, Ijzermans CJ (2003). The influence of COPD on health-related quality of life independent of the influence of comorbidity. J Clin Epidemiol..

[28] Okamoto LJ, Noonan M, DeBoisblanc BP, Kellerman DJ (1996). Fluticasone propionate improves quality of life in patients with asthma requiring oral corticosteroids. Ann Allergy Asthma Immunol..

[29] Wallace RJ, Brown BA, Griffith DE, Girard WM, Murphy DT, Onyi GO (1994). Initial clarithromycin monotherapy for Mycobacterium avium-intracellulare complex lung disease. Am J Respir Crit Care Med..

[30] Hasegawa N, Nishimura T, Ohtani S, Takeshita K, Fukunaga K, Tasaka S (2009). Therapeutic effects of various initial combinations of chemotherapy including clarithromycin against Mycobacterium avium complex pulmonary disease. Chest..

[31] Jeong BH, Jeon K, Park HY, Kim SY, Lee KS, Huh HJ (2015). Intermittent antibiotic therapy for nodular bronchiectatic Mycobacterium avium complex lung disease. Am J Respir Crit Care Med..

[32] McHorney CA, Ware JE, Lu JF, Sherbourne CD (1994). The MOS 36-item Short-Form Health Survey (SF-36): III. Tests of data quality, scaling assumptions, and reliability across diverse patient groups. Med Care.

[33] Morimoto K, Iwai K, Uchimura K, Okumura M, Yoshiyama T, Yoshimori K (2014). A steady increase in nontuberculous mycobacteriosis mortality and estimated prevalence in Japan. Ann Am Thorac Soc..

[34] Ridker PM (2001). High-sensitivity C-reactive protein: potential adjunct for global risk assessment in the primary prevention of cardiovascular disease. Circulation..

[35] Jayakumar A, Vittinghoff E, Segal MR, MacKenzie WR, Johnson JL, Gitta P (2015). Serum biomarkers of treatment response within a randomized clinical trial for pulmonary tuberculosis. Tuberculosis (Edinb).

[36] Shivakoti R, Yang WT, Gupte N, Berendes S, Rosa AL, Cardoso SW (2015). Concurrent anemia and elevated C-reactive protein predicts HIV clinical treatment failure, including tuberculosis, after antiretroviral therapy initiation. Clin Infect Dis..

[37] Liu X, Mayes MD, Pedroza C, Draeger HT, Gonzalez EB, Harper BE (2013). Does C-reactive protein predict the long-term progression of interstitial lung disease and survival in patients with early systemic sclerosis?. Arthritis Care Res (Hoboken).

